# REMODEL: Rethinking Deep CNN Models to Detect and Count on a NeuroSynaptic System

**DOI:** 10.3389/fnins.2019.00004

**Published:** 2019-02-22

**Authors:** Rohit Shukla, Mikko Lipasti, Brian Van Essen, Adam Moody, Naoya Maruyama

**Affiliations:** ^1^Department of Electrical and Computer Engineering, University of Wisconsin-Madison, Madison, WI, United States; ^2^Lawrence Livermore National Laboratory, Livermore, CA, United States

**Keywords:** deep learning, convolutional neural network, IBM TrueNorth Neurosynaptic System, neuromorphic computing, spiking neural network, aerial image analysis

## Abstract

In this work, we perform analysis of detection and counting of cars using a low-power IBM TrueNorth Neurosynaptic System. For our evaluation we looked at a publicly-available dataset that has overhead imagery of cars with context present in the image. The trained neural network for image analysis was deployed on the NS16e system using IBM's EEDN training framework. Through multiple experiments we identify the architectural bottlenecks present in TrueNorth system that does not let us deploy large neural network structures. Following these experiments we propose changes to CNN model to circumvent these architectural bottlenecks. The results of these evaluations have been compared with caffe-based implementations of standard neural networks that were deployed on a Titan-X GPU. Results showed that TrueNorth can detect cars from the dataset with 97.60% accuracy and can be used to accurately count the number of cars in the image with 69.04% accuracy. The car detection accuracy and car count (–/+ 2 error margin) accuracy are comparable to high-precision neural networks like AlexNet, GoogLeNet, and ResCeption, but show a manifold improvement in power consumption.

## 1. Introduction

Neural networks today are achieving state-of-the-art performance in competitions across a range of fields. Recent advances in deep learning (LeCun et al., 2015) have motivated the development of neural hardware substrates that are tailored to implementing deep networks with extremely low power and efficiency for a variety of embedded systems applications. Hardware that mimics the computational capabilities of a human brain through spiking neural networks has been shown to be not only extremely energy-efficient, but also capable of scaling up to large neural networks. Examples include the IBM TrueNorth Neurosynaptic System (Merolla et al., 2014), SpiNNaker (Furber et al., 2014), and the BrainScaleS project (Schemmel et al., 2008), all of which mimic the computational behavior of spiking neurons and can also be used to deploy deep neural networks.

One of the major challenges that these spiking neural network-based platforms faced was deploying convolutional neural networks (CNNs) on spiking neurons. This issue was addressed in the recent work from Cao et al. (2015) and Esser et al. (2016), and Eta Compute (Moore, 2018). The authors in Esser et al. (2016) have proposed an algorithm named energy-efficient deep neuromorphic networks (EEDN) to map CNNs on TrueNorth. EEDN networks achieved at or near state of the art accuracy when compared with traditional 32-bit precision neural networks on standard benchmarks and they operated at a much higher throughput (Frames Per Second) per watt. These promising results show potential for deploying spiking neural network based platforms for a variety of applications where battery life and power consumption are primary concerns. Such applications include video surveillance, UAV surveillance, aerial image analysis, etc.

Prior work such as Esser et al. (2015, 2016), Wen et al. (2016), Rueckauer et al. (2017), and Sengupta et al. (2018) have discussed about how to efficiently train neural network models so that the inference neural network can be easily mapped onto low precision hardware such as TrueNorth without any loss in output accuracy. But these prior works have only done the evaluations against small object recognition datasets such as MNIST, CIFAR-10, and CIFAR-100.

**Figure 1:**
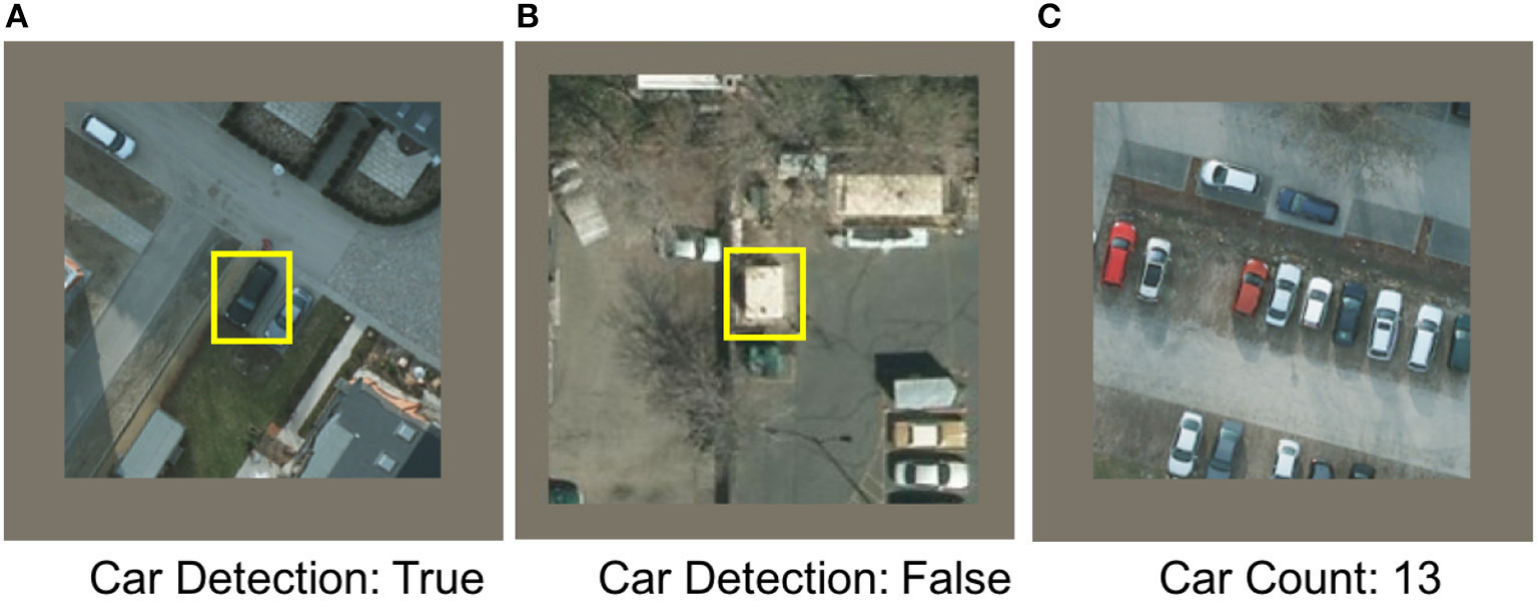
Sample images from COWC dataset (Mundhenk et al., 2016). Images are 192-by-192 pixels. For detection, **(A,B)**, the model's goal is to detect whether a car is present in the center 48-by-48 pixels or not. Even though there are cars present in **(B)**, the label has been set to false because there is no car in the center 48-by-48 pixels of the image. For the counting task, **(C)**, the goal is to count the exact number of cars present in an image. The example shown in the figure has the label value “13,” since there are 13 cars in the image.

**Figure 2:**
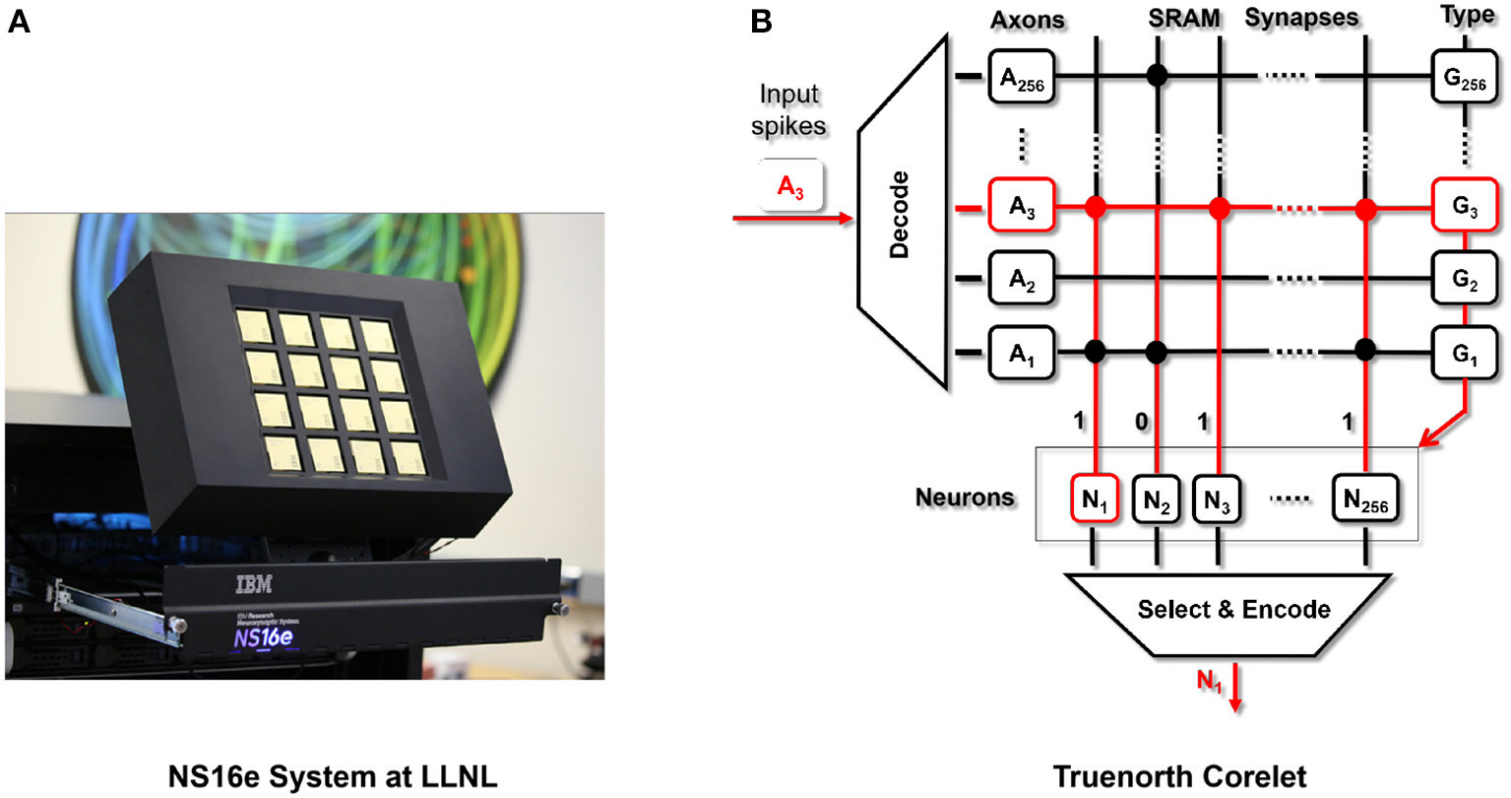
**(A)** NS16e hardware system that was developed by IBM (Image from Shah, 2016). **(B)** Single neurosynaptic core which forms the computational block of the TrueNorth chips with the details presented in Cassidy et al. (2013) and Nere (2013).

Prior work never listed out the challenges that might occur when mapping large CNN or DNN structures on TrueNorth for bigger datasets with large annotated images. For bigger datasets resource limitations and the CNN model limitations that TrueNorth can support start becoming a bottleneck. In this paper we evaluate the challenges related to deployment of EEDN trained neural network on TrueNorth hardware. Discussions that have been reported in this article are meant to complement the opportunities and challenges for spiking neural network hardware that have been reported in Pfeiffer and Pfeil (2018). The evaluations have been done against publicly-available dataset of overhead aerial images of cars that was proposed by Mundhenk et al. (2016) (Henceforth referred as COWC dataset). Examples from COWC dataset have been shown in Figure 1. As the neural network structures start becoming more complex, we have to keep in mind limited number of TrueNorth (Henceforth referred as TN) cores that are available and design a neural network structure so that we can obtain benefits by using hardware substrates more judiciously. This paper presents design decisions that a developer would have to make to design a neural network for the TrueNorth NS16e system (Sawada et al., 2016) that is shown in **Figure 2**. The goal of this work is to present how knowledge of hardware architecture affects the decisions and parameter choices made while training and deploying neural networks on TrueNorth. These observations can assist us in maximizing the benefits of TrueNorth's available hardware computational resources.

Contributions of the research proposed in this paper are:

- Evaluate TrueNorth deployed CNNs for counting and detection tasks on COWC dataset (Mundhenk et al., 2016).
- Resources consumed by AlexNet (Krizhevsky et al., 2012) and VGG-16 (Simonyan and Zisserman, 2014) neural networks when deployed on NS16e hardware (Sawada et al., 2016). Identifying the architectural bottlenecks of these CNN structures and proposed changes to the CNN structure so that it could be deployed on NS16e hardware.
- Analysis of change in resource consumption and output accuracy based on the prior works such as, network-in-network structure (Lin et al., 2013), MobileNets (Howard et al., 2017), and YOLO (Redmon and Farhadi, 2016) neural network models.
- Discussions presented in section 4 outline the opportunities that are present in SNN hardware that can address the challenges present in TrueNorth architecture and EEDN training algorithm.

## 2. Materials and Methods

### 2.1. Background

#### 2.1.1. Cars Overhead With Context Dataset

Paraphrasing the work presented by Mundhenk et al. (2016), the cars overhead with context (COWC) data set is a large set of annotated overhead aerial images that contain cars. This dataset is useful for training Deep Neural Networks (DNNs) so that they are able to perform area based surveillance by detecting and counting cars that are present in the image. This dataset could be potentially used to keep track of volume of cars by deploying the trained DNNs on unmanned aerial vehicles or drones. The goal of this dataset is to allow DNNs to determine the relationship between context and appearance such that something that looks very much like a car is detected even if it is in an unusual place. Unlike datasets such as MNIST, CIFAR-10, and CIFAR-100, where the maximum image size for which the neural network models were trained was 64-by-64 pixels (Esser et al., 2015, 2016), the COWC dataset consists of annotated images of size 192-by-192 pixels and this dataset requires us to solve a regression problem (counting the number of cars present in the entire image).

Figure 1 shows some of the sample images from the dataset. The goal of our work is to map this problem onto a low-power neural network architecture such as TrueNorth and evaluate its performance. The images in this dataset cannot be cropped out for training because the labels have been set for the entire image. For example, if the image shown in Figure 1C was cropped out for training, then the label “13” won't be correct, because the cropped out piece of image won't have the same number of cars as the label.

#### 2.1.2. NS16e System

Summarizing the details of TrueNorth, as presented in Sawada et al. (2016), a single chip consists of 4,096 neurosynaptic cores (as shown in Figure 2B), tiled as a 64 × 64 array. Neurons integrate incoming spikes weighted by the synaptic strength and when a neuron membrane potential integrates beyond its threshold, it fires a spike, transmitting it to a target axon on any core in the network. In the same clock tick when neuron fired, the neuron would reset its membrane potential. Truenorth chips can be scaled beyond a single chip using SerDes links. As a result it is relatively simple to tile TrueNorth chips in a two-dimensional array, enabling the NS16e scale-up system.

**Figure 3:**
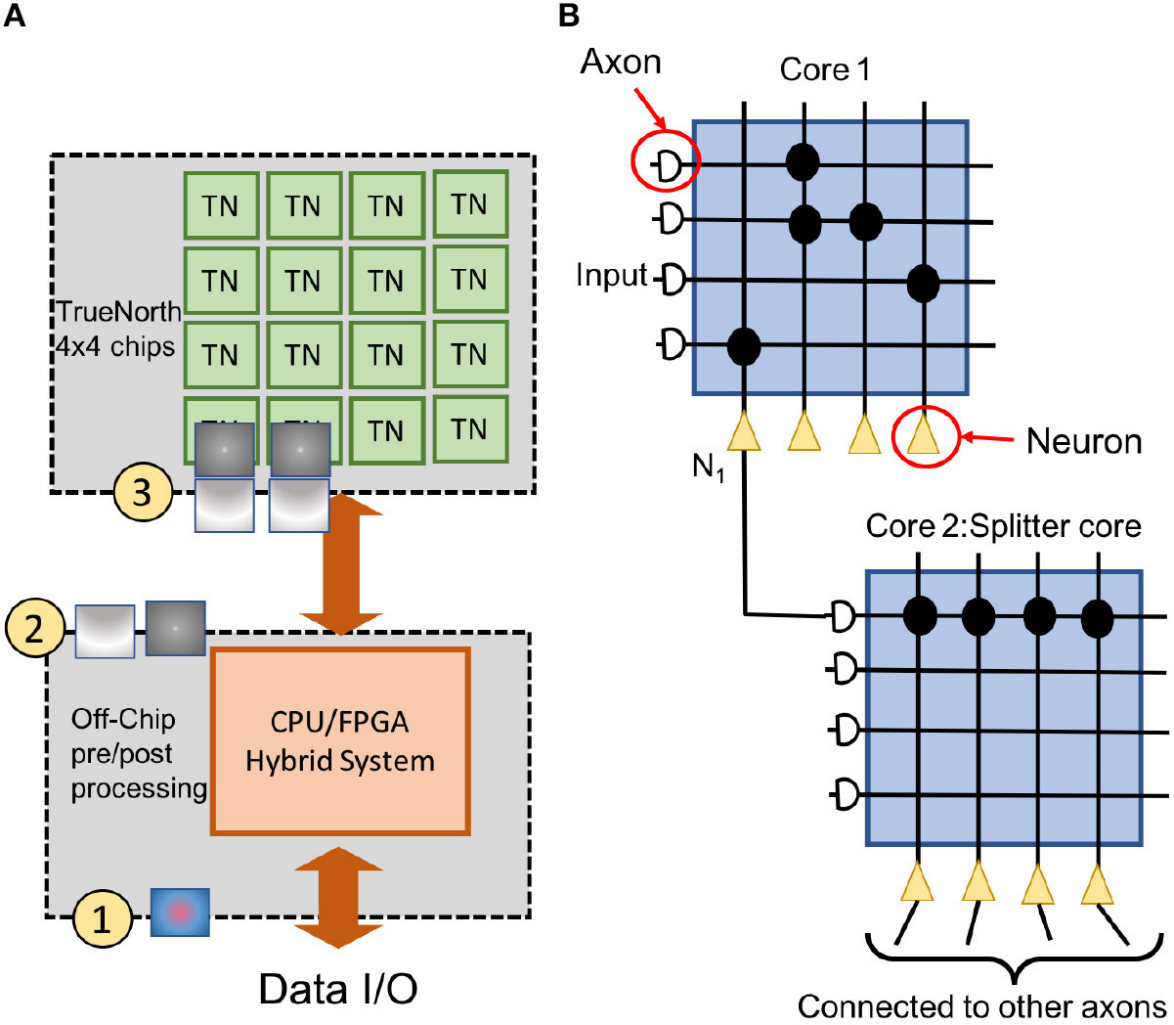
The figure describes the NS16e system setup. **(A)** NS16e system consists of three stages. The hybrid CPU/FPGA system performs data pre/post processing and image binarization. The computed spikes are later sent to TN chips on which the CNN has been deployed **(B)** An image of how splitters are used on TrueNorth for increasing a neuron's fan-out.

Figure 3 shows a high-level setup for NS16e system and, the flow of computations happens between the off-chip system and NS16e hardware. In TrueNorth (as shown in Figure 3A) image binarization (data transduction) happens outside the TN chips, that is, in the CPU/FPGA hybrid system. 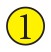 When an RGB image is fed to the TrueNorth system, 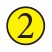 based on the learned convolutional layer weights and output feature count of the transduction layer, a corresponding number of binary images is produced. 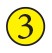 These binary images are then sent to TN chips and on these TN chips these image features are fanned out using splitters (Figure 3B) so that multiple filter weights can operate in parallel on the same set of binary image features.

### 2.2. CNN Design Decisions

In this section we present design decisions for modifying standard neural network structures for NS16e hardware platform. First we will understand different set of computations that happen in standard neural network architectures, followed by what are the resource or architectural bottlenecks that we face when mapping these standard neural network architectures. Once we have understood the challenges and the architectural bottlenecks, we will look at how these issues can be addressed by proposing different neural network structure design.

#### 2.2.1. Formulate Regression Problem as a Classification Problem

To maintain high throughput, TrueNorth performs operations in stream of single bits. A trained TrueNorth network will have ternary weights {–1,0,1} and binary activation {0,1}; as a result, algorithms that require us to solve regression problems, i.e., infer continuous output values, such as the car count in the image, present a challenge. Being able to estimate high precision values by using binary activation functions is a hard problem. In the context of TrueNorth and spiking neural networks, prior work such as Diehl et al. (2016) and Shukla et al. (2017, 2018) have represented regression output values using rate coding scheme, where the expected value of spike train over a time window represented the value. But with this scheme the operating frequency of hardware starts becoming the bottleneck. To match the biological clock rate TrueNorth operates at 1 KHz frequency (Akopyan et al., 2015); as a result, if the problem requires us to estimate continuous numbers, we would have to count the number of spikes received over a window of time to estimate the output and this ends up slowing down the computation time. We can circumvent this issue by recasting the regression problem as a classification problem with estimated discrete values as outputs. This approach might require more hardware neurons for a large number of output bins. For the dataset that we are studying, the car counting problem would predict from 65 classes. As noted in Mundhenk et al. (2016), the number of cars in each image patch lie in the interval between 0 to 64.

#### 2.2.2. Case Study: Map AlexNet Neural Network Model Onto TrueNorth

We will start off the discussion by mapping AlexNet neural network model onto TrueNorth NS16e hardware. The accuracy and hardware analysis of AlexNet-TrueNorth model has been presented in Table 1.

**Table 1:** Convolutional neural network structure analysis and testing accuracy.

**Model name**	**Detection** ** accuracy (in %)**	**Counting accuracy (in %)**	**Chips required** ** for first 3 TN** ** CNN layers**
AlexNet (Figure 4A)	97.62	67.97	N/A
AlexNet modified (Figure 5A)	89.98	48.82	3.19
VGG-16 modified (1) (Figure 8A)	96.09	67.96	8.67
VGG-16 modified (2) (Figure 8B)	97.25	67.82	8.67
Deeper CNN structure 1 (Figure 11A)	97.52	68.21	11.16
Deeper CNN structure 2 (Figure 11B)	97.60	69.04	11.16

**Figure 4:**
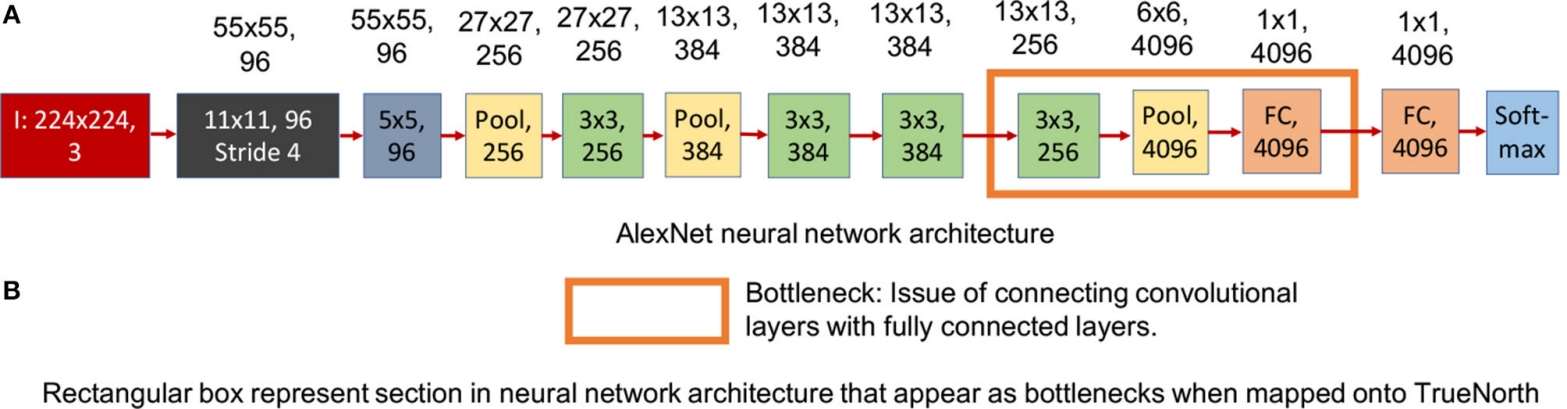
This figure shows the standard AlexNet neural network architecture. The numbers written on top of the blocks show the output feature dimension of that block in CNN model. **(A)** Shows the standard AlexNet neural network model (Krizhevsky et al., 2012). **(B)** Sections in the standard AlexNet neural network structure that pose a problem when trying to map it onto TrueNorth.

**Figure 5:**
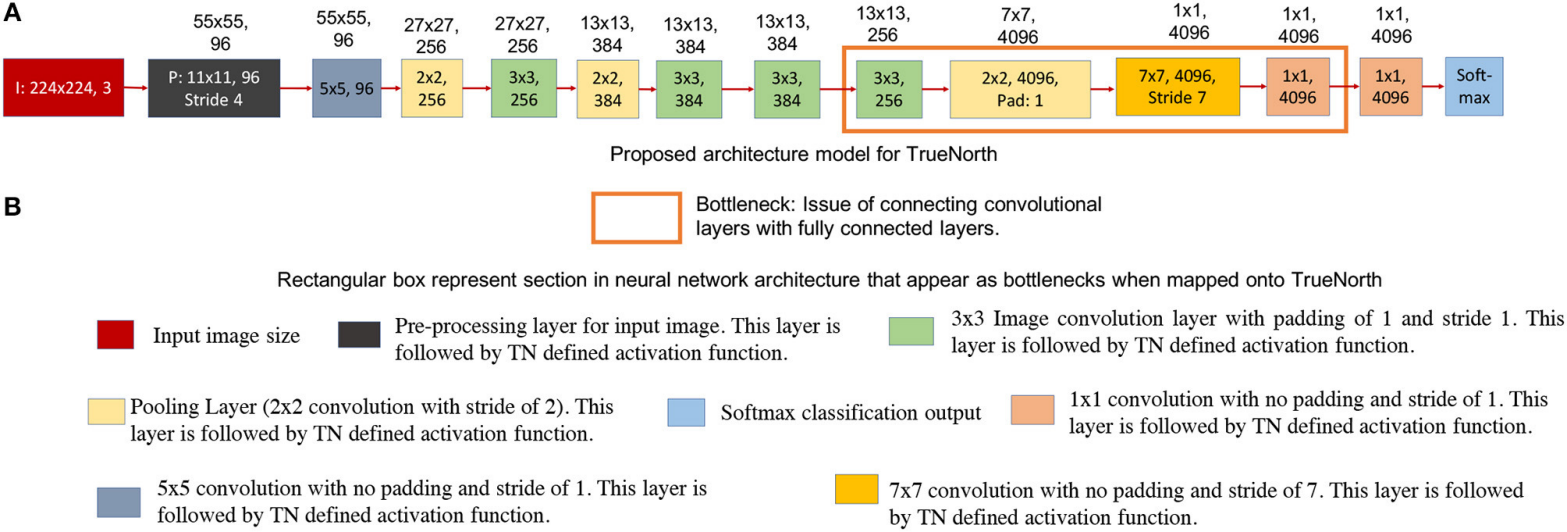
This figure shows the AlexNet implementation on TrueNorth. The numbers written on top of the blocks show the output feature dimension of that block in CNN model. **(A)** Shows the modified AlexNet architecture for TrueNorth implementation. **(B)** Sections in the modified AlexNet neural network structure there later fixed when trying to map the standard AlexNet onto TrueNorth. The output feature dimensions of 9th CNN layer in the proposed modified AlexNet is different for standard AlexNet model (Figure 4). This is because the 8th CNN layer in this modified layer has a padding of 1, unlike the standard AlexNet mode where the 8th CNN layer did not have any padding.

Figure 4A shows the neural network model of a standard AlexNet structure and Figure 5A shows the modified AlexNet neural network model for TrueNorth ns16e hardware. The difference between the neural network is highlighted using the rectangular box as described in Figures 4B, 5B. As shown in Esser et al. (2016), Equation (1) defines the activation function used by CNN layers that are deployed on TN.

(1) $$\text{TN defined activation function}=\{\begin{array}{l} 1\;\text{neuron filter response}\geq \text{0}\\ 0\;\text{otherwise} \end{array}$$

##### 2.2.2.1. Challenges with AlexNet neural network model

In TrueNorth, neural network architectures where a large set of convolutional network neurons need to be connected to fully connected layers will consume a considerable amount of hardware resources. Thus, the proposed CNN avoid fully-connected layers, and instead the convolutional features are progressively downsampled to a one-by-one convolution. For example, in AlexNet (Krizhevsky et al., 2012), there are 9,216 neurons that present the output features of the 5th convolutional layer and these have to be connected to 4,096 neurons present in first fully connected layer. This kind of structure is crucial for datasets where we have to scan through the entire image pixels before predicting an output, such as counting the number of cars in our experiments. Prior work done by the authors have used either only a convolutional neural network structure (Esser et al., 2016) or just a fully connected neural network (Esser et al., 2015) in the context of object recognition. Earlier work have not addressed how to interface convolutional to fully connected layers. Mapping such CNN outputs on TrueNorth would require us to connect each convolutional layer neuron to all neurons in the fully connected layer. As a result, we might either end up using large number of cores as splitters to implement this fanout, as shown in Figure 3B, or we might use additional hardware resources to rearrange the 3D convolutional layers for a 1D fully connected layer.

##### 2.2.2.2. Proposed modification for AlexNet neural network model

We have addressed the challenges associated with convolutional layer and fully connected layer connections by downsampling the CNN output all the way down to a one-by-one convolution using strided convolutions. The downsampling has been performed by having a convolutional layer that has convolution window of size 7 x 7 pixels and a stride of 7, as shown by the rectangular box in Figure 5A. Similar downsampling has been used in MobileNets (Howard et al., 2017). This structure ensures that the output layer considers the entire image but is more friendly to TrueNorth's limited fanout capability. The proposed AlexNet Figure 5A requires **9 TN chips** for deployment onto NS16e hardware.

Readers should observe that the output feature dimensions of 9th CNN layer is different for standard AlexNet model (Figure 4) and modified AlexNet model (Figure 5A). This is because the 8th CNN layer in this modified layer has a padding of 1, unlike the standard AlexNet model where the 8th CNN layer did not have any padding.

#### 2.2.3. Case Study: Map VGG-16 Neural Network Model Onto TrueNorth

Next we will look at the challenges that come up when we map VGG-16 style architecture onto the TrueNorth ns16e hardware. As explained earlier, Equation (1) defines the activation function used by CNN layers deployed on TN.

**Figure 6:**
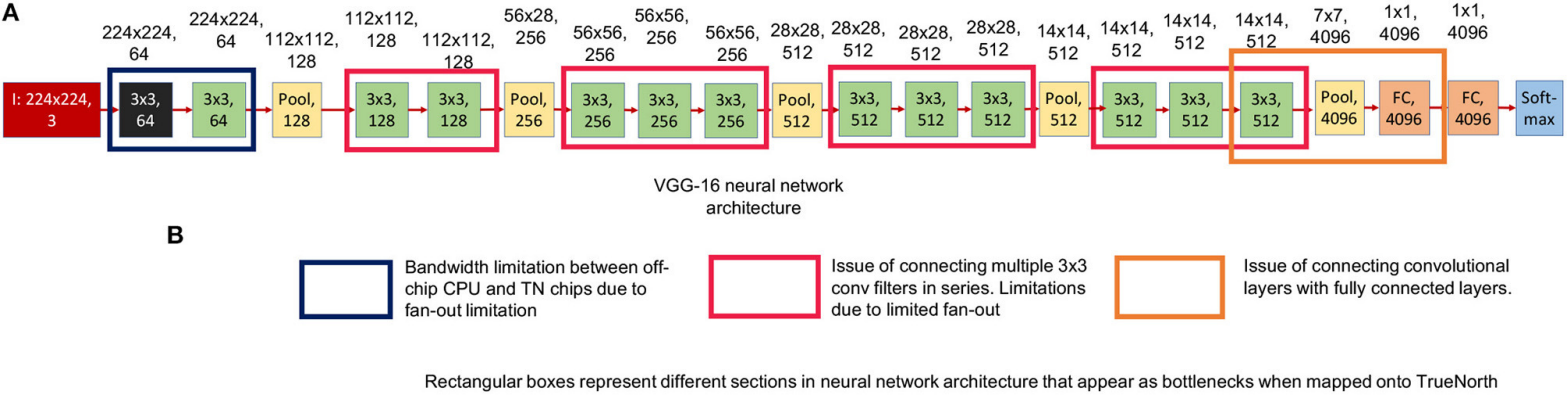
This figure shows the standard VGG-16 neural network architecture implementation. The numbers written on top of the blocks show the output feature dimension of that block in CNN model. **(A)** Shows the standard VGG-16 neural network model (Simonyan and Zisserman, 2014). **(B)** Three sections in the standard VGG-16 neural network structure that pose a problem when trying to map it onto TrueNorth.

Figure 6A shows the neural network model of a standard VGG-16 structure. Three different sections of VGG-16 neural network structure that pose a problem for TrueNorth implementation have been highlighted using the rectangular box in Figure 6B.

**Figure 7:**
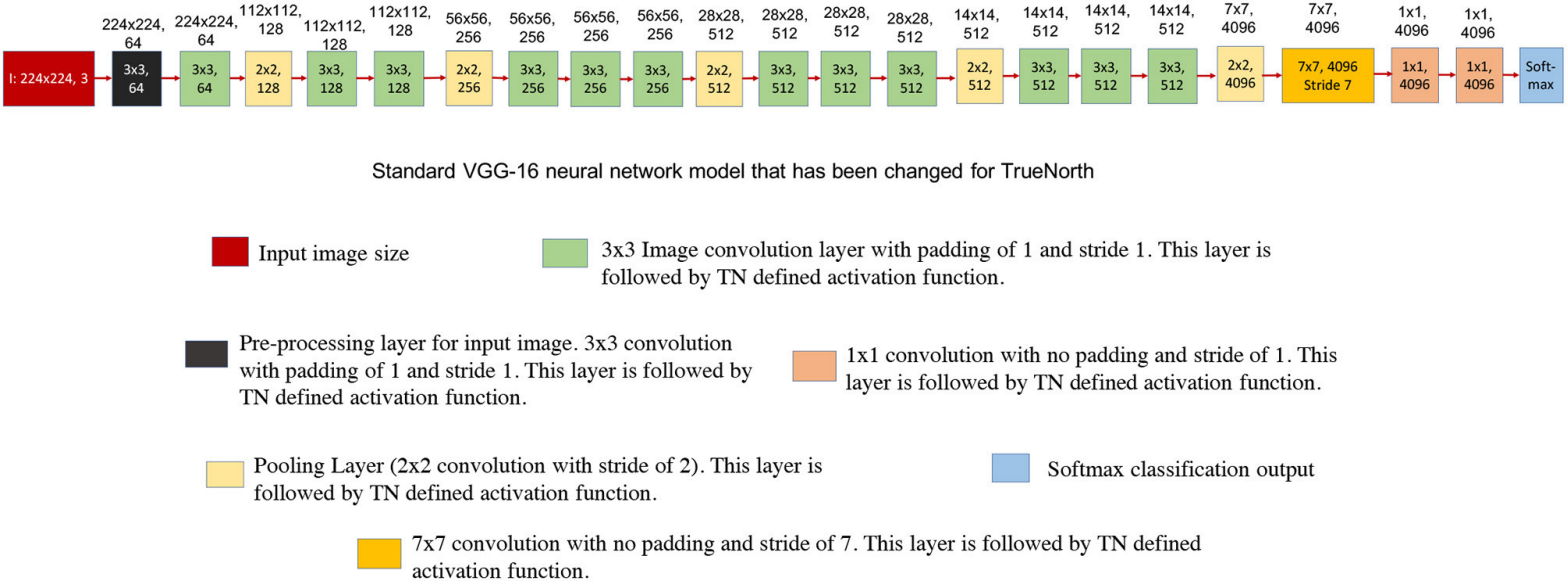
This figure shows the standard VGG-16 neural network architecture that has been modified for TrueNorth implementation. The numbers written on top of the blocks show the output feature dimension of that block in CNN model. Similar to AlexNet, this standard VGG-16 neural network model has CNN features that have been downsampled all the way down to a one-by-one convolution using convolution kernels of size 7 x 7 and stride of 7.

Figure 7 shows the standard VGG-16 neural network architecture that has been modified for TrueNorth implementation. Similar to AlexNet, this standard VGG-16 neural network model has CNN features that have been downsampled all the way down to a one-by-one convolution using convolution kernels of size 7 x 7 and stride of 7.

##### 2.2.3.1. Challenges in VGG-16: hardware resource limitation

If the users were to map the standard VGG-16 neural network model that has been shown in Figure 7, then the EEDN trained CNN model would require more than **49 TrueNorth chips** to deploy the said neural network; whereas, NS16e hardware has only 16 available TN chips. It is important for us to understand the architectural bottlenecks in the NS16e hardware that does not allow us to map the VGG-16 neural network structure and how can it be addressed when designing a neural network model for an application.

##### 2.2.3.2. Challenges in VGG-16: input feature size and feature count

**Figure 8:**
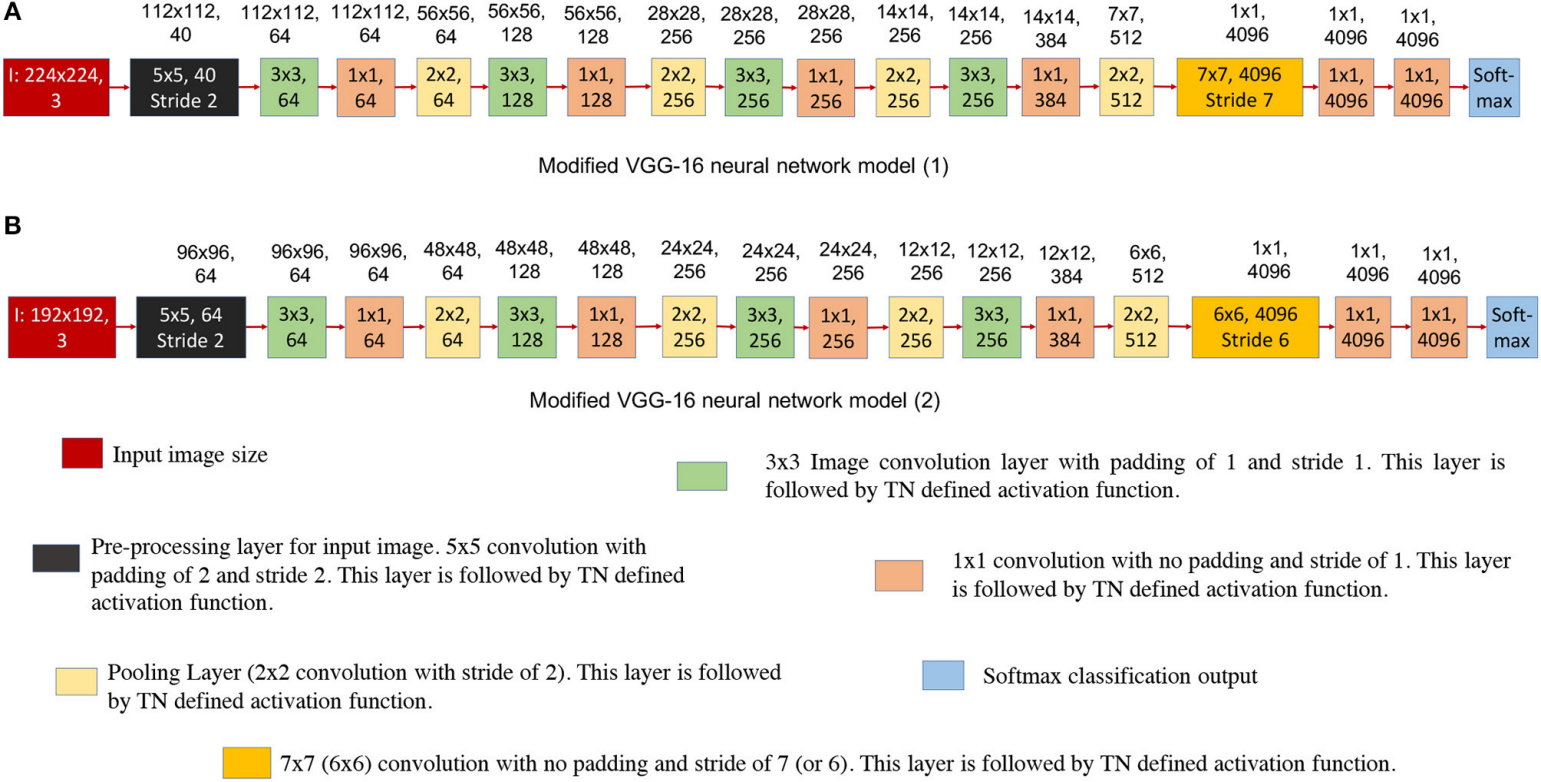
This figure shows the modified VGG-16 neural network architecture for TrueNorth ns16e hardware. The numbers written on top of the blocks show the output feature dimension of that block in CNN model. **(A)** Shows the modified VGG-16 neural network model (1) where the input image size if kept at 224x224 pixels. **(B)** Shows the modified VGG-16 neural network model (2) where the input image size if kept at 192x192 pixels.

In TrueNorth (as shown in Figure 3A) image binarization (data transduction) happens outside the TN chips, that is, in the CPU/FPGA hybrid system. As discussed in section 2.1.2, step 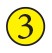, the binary image features representation are fanned out inside TN chips, thus, a considerable amount of resources are taken up by splitters for this pixel fan-out. That is, neurons that could have been potentially used for computation, have to be utilized as resources that would create multiple copies of the input features so that different convolutional filters can operate on these input features in parallel. Since prior work (Esser et al., 2015, 2016) have trained neural networks for a maximum input image size of 64-by-64 pixels, this problem of fan-out becomes more significant if the dataset has larger image size (192-by-192 pixels in case of COWC dataset). To minimize the fan-out resource utilization we have to either reduce the image size or reduce the number of input features. Next section will explain the reduction in required hardware resources for fan-out with the modified VGG-16 architecture (Figure 8). A more thorough analysis on the trade-off between fan-out requirement and, different input features and smaller input image sizes, has been presented in section 3.2.

##### 2.2.3.3. Proposed modification for VGG-16 input

**Figure 9:**
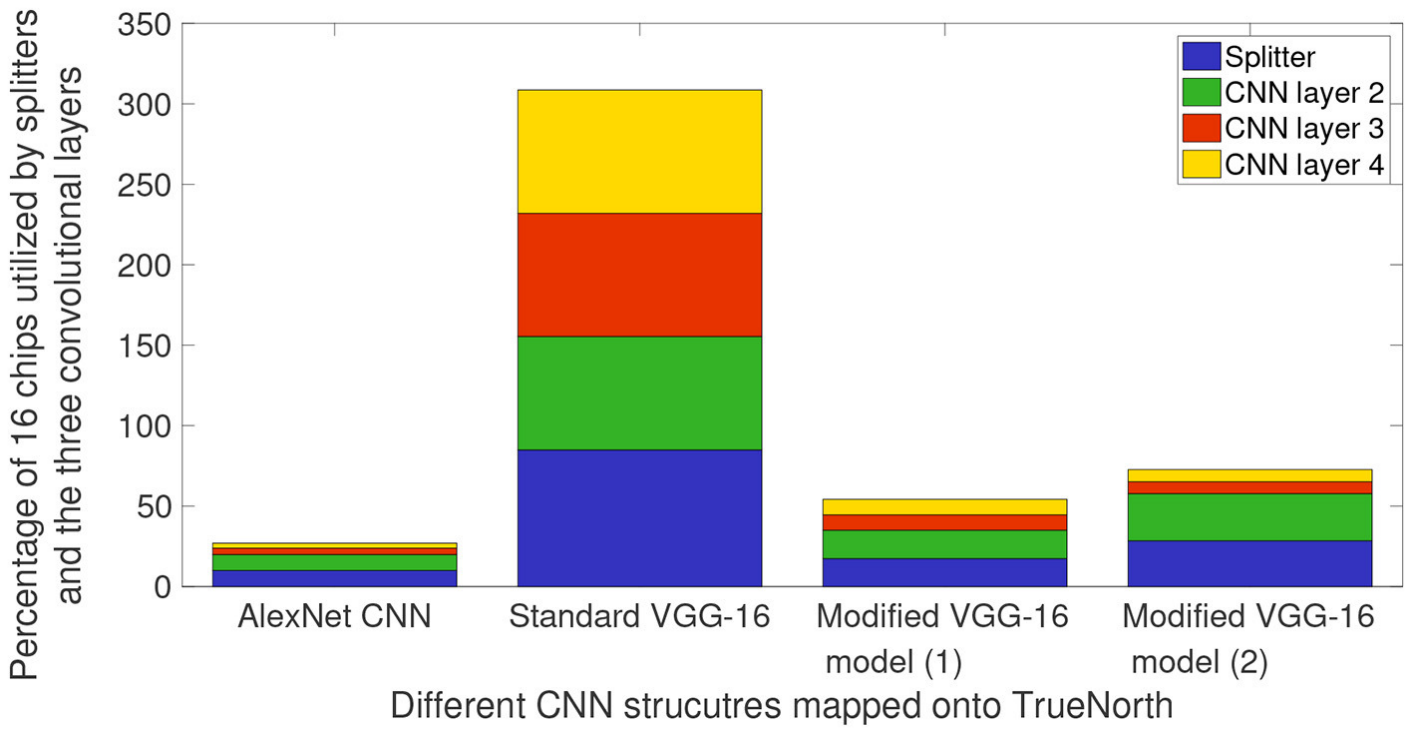
Percentage of TN chips required on NS16e system for splitters and the three CNN layers that are deployed on the hardware. These chip consumption values are for AlexNet CNN presented in Figure 5, VGG-16 CNN models that have been presented in Figures 7, 8.

Figures 8A,B show the modified neural network models of VGG-16 structure and Figure 9 shows the hardware requirements for mapping CNN layers on TrueNorth. For understanding the hardware resource consumption, we focus on the TN chips required by first three layers of CNNs deployed on TN and splitters. Figure 8A keeps the input image size same as the one for standard VGG-16 structure, but the number of features in the initial layer had to be reduced from 64 to 40. This is because having a feature count of 64 for the first layer requires 14 chips just to handle the fan-out using splitters. By reducing the number of feature count to 40, TrueNorth requires 3 chips for fan-out. Similarly, the fan-out constraints can be addressed by reducing the input image size as shown in Figure 8B. Here the goal was to keep the number of features in the initial layer to be 64, same as the one standard VGG-16 structure. To achieve this we have proposed an input image of comparatively smaller size, that is, instead of having an image of size 224 x 224 pixels, we have an input image of size 192 x 192 pixels. As explained earlier (section 2.1.1), the COWC dataset has images of size 192 x 192 pixels. Therefore, by having a comparatively smaller images as input we do not sacrifice any pixel level information, but after this modification we require only 5 TN chips to serve as splitters.

Figure 9 shows the breakdown of chip utilization for the splitters, and convolutional layers 2, 3, and 4, since these four layers consumed the most number of hardware resources. It can be inferred from Figure 9 that by having small input feature size, TN requires significantly less number of hardware resources for splitters and the first CNN layer that is deployed on TN. AlexNet downsamples the input images by having a CNN layer of stride 5 in the initial layer. Whereas, for VGG-16 models, the user would have to keep in mind the input feature count and input image size because the initial layer has CNN layer of stride 2.

##### 2.2.3.4. Challenges in VGG-16: size of convolutional kernels

Selecting an appropriate convolutional kernel size is crucial for deploying CNNs on a hardware constrained substrate. Hence, smaller convolutional kernel would be very helpful. TrueNorth convolutional layers support 1 x 1 convolutions that were proposed by Lin et al. (2013). The pooling layers in EEDN networks have been implemented as convolutional operations with a stride of 2, as proposed by Springenberg et al. (2014). Larger kernels such as 5 x 5 kernels are good for learning higher level features in an image, whereas smaller kernels such as 3 x 3 and 1 x 1 kernels are good for learning lower level features and 1 x 1 convolutions can add non-linearity at a pixel level of the image. These convolution operations tend to learn the object properties and give prediction results based on these properties.

##### 2.2.3.5. Proposed method for selecting kernel size

Convolution kernels that are bigger than 3 x 3, are used only in the preprocessing layers. As presented in section 2.1.2 and Figure 3A, image binarization or preprocessing happens off-chip. As a result, even if larger convolutional kernels are selected for the first CNN layer, TrueNorth resources do not get consumed because the first layer (or preprocessing layer) gets implemented off-chip. Therefore, as shown in Figure 8, the first CNN layer of modified VGG-16 structure has convolutional kernels of size 5 x 5 pixels and this layer is implemented off-chip. Similarly, we were able to have convolutional kernels of size 11 x 11 for the first CNN layer in modified AlexNet model as shown in Figure 5A. On the other hand, rest of the CNN layers have smaller sized convolutional kernels, that is, the convolutional kernels are of size 3 x 3 or 1 x 1. Smaller kernels require fewer computational resources, enabling us to fit a denser and wider network on the TrueNorth substrate. The 1 x 1 convolution layers require 9 times fewer groups than the 3 x 3 layers and 25 times fewer groups than the 5 x 5 layers. A similar idea of having only 1 x 1 and 3 x 3 convolution layers in the CNN structure was proposed by the authors of SqueezeNet (Iandola et al., 2016).

**Figure 10:**
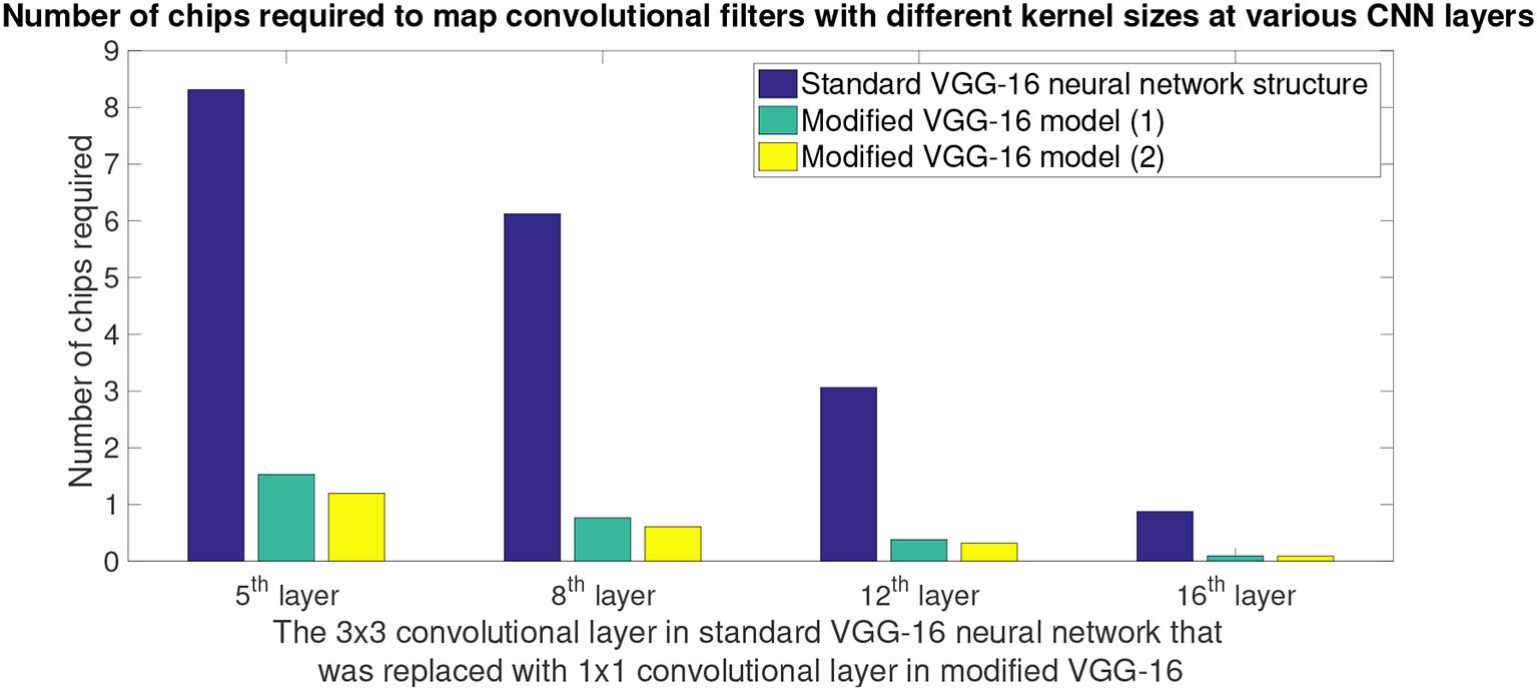
Hardware savings that is achieved by replacing 3 x 3 convolution kernels in standard VGG-16 model with 1 x 1 convolution kernels. Modified VGG-16 model (1) refers to the CNN structure presented in Figure 8A, and modified VGG-16 model (2) refers to the CNN structure presented in Figure 8B. X-axis shows the convolutional layer in standard VGG-16 (Simonyan and Zisserman, 2014) CNN that originally had 3 x 3 convolution kernel, but they were replaced by 1 x 1 kernels in the modified VGG-16 (Figure 8) model for NS16e. Y-axis shows the number of chips that were consumed by the CNN layer when deployed onto NS16e.

Figure 10 shows a comparison between hardware resources required by replacing certain 3 x 3 convolutions in standard VGG-16 neural network structure with 1 x 1 convolutions. Note that the x-axis of plot in Figure 10 shows the CNN layer in standard VGG-16 that were replaced with 1 x 1 convolution kernels. 5th convolution layer of standard VGG-16 corresponds to 3rd convolution layer of modified VGG-16 structures; similarly 8th convolution layer of standard VGG-16 corresponds to 6th convolution layer of modified VGG-16 structures, 12th convolution layer of standard VGG-16 corresponds to 9th convolution layer of modified VGG-16 structures and 16th convolution layer of standard VGG-16 corresponds to 12th convolution layer of modified VGG-16 structures. It can be observed from the plots that by having smaller convolutional kernels, modified VGG model (1) (Figure 8A) is able to achieve up to 6.6x reduction in hardware resources; similarly modified VGG model (2) (Figure 8B) is able to achieve up to 8.3x hardware resources. Note that the second modified VGG model is performing computations on comparatively smaller image patches, as a result, it requires less number of hardware resources when compared with all of the other neural network structure models.

##### 2.2.3.6. Discussion on fully convolutional neural network of VGG-16

As presented in section 2.2.2, one of the challenges that users might face when mapping standard neural network structures onto TrueNorth is that currently the proposed hardware architecture does not support convolutional layer to fully connected layer connections. Similar to modified AlexNet model, while mapping VGG-16 onto TrueNorth, the CNN features are downsampled all the way down to a one-by-one convolution using strided convolutions. The downsampling has been performed by having a convolutional layer that has convolution window of size 7 x 7 pixels and a stride of 7, (as shown in Figure 8A) or by having a convolutional layer that has convolution window of size 6 x 6 pixels and a stride of 6 (as shown in Figure 8B).

#### 2.2.4. Case Study: Deeper Fully Convolutional Neural Network

**Figure 11:**
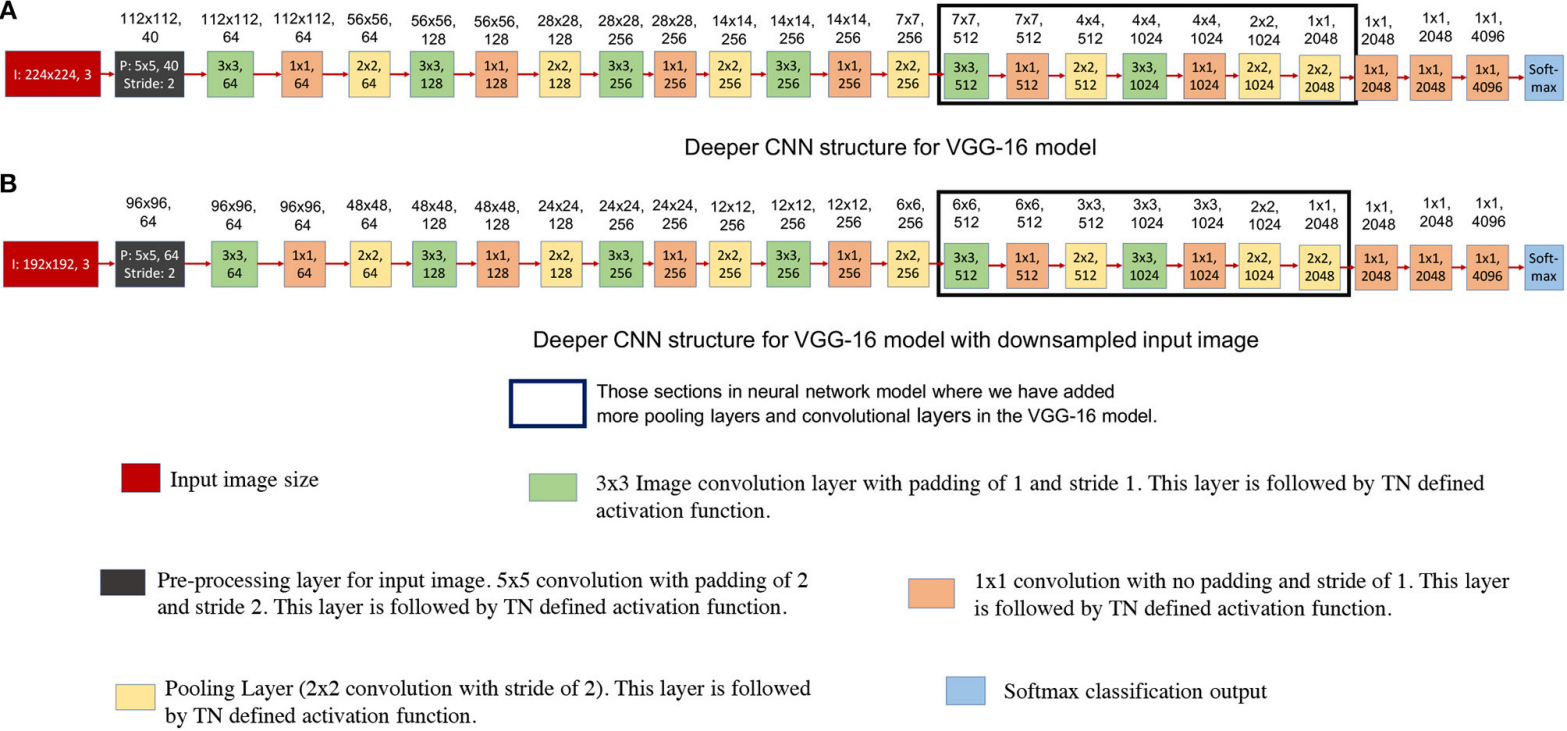
This figure shows deeper convolutional neural network architecture for TrueNorth ns16e hardware. The numbers written on top of the blocks show the output feature dimension of that block in CNN model. These CNN models are extensions of the VGG-16 models that were proposed in Figure 8. **(A)** shows the deep convolutional neural network model where the input image size is kept at 224x224 pixels. **(B)** shows the deep convolutional neural neural network model where the input image size is kept at 192x192 pixels.

As we have discussed in earlier designs, TrueNorth does not support convolutional layer to fully connected layer connections. The proposed solutions for the earlier neural network designs were to downsample intermediate CNN features all the way down to a one-by-one convolution using strided convolutions. We achieved this by taking average of CNN features that are of size 7 x 7 pixels (as shown in Figures 5A, 8A) or 6 x 6 pixels (as shown in Figure 8B). In this section we propose having a deeper fully convolutional neural network for modified VGG-16 network (that were earlier shown in Figure 8). Unlike the proposed previous two designs, the CNN features are downsampled all the way down to a one-by-one convolution using additional strided convolutions of size 2 x 2 instead of having convolutional filters of size 7 x 7 or 6 x 6. The deeper convolutional neural network has been shown in Figure 11. The proposed deep CNN model does not require any additional TrueNorth chips for deployment. Since the image size has become significantly small, we do not observe any significant change in hardware requirements. As a result, the proposed deep CNN model can be mapped using all of the 16 TrueNorth chips that are available on NS16e hardware.

## 3. Results

This section describes how the decisions that have been proposed in section 2.2 affect accuracy and hardware resource utilization. The EEDN-trained CNN structures have been compared against more standard neural network models that were deployed on Titan X GPU. All of the neural networks were trained only for COWC dataset. For EEDN trained CNNs the output layer has a *softmax* loss function. The car detection dataset had two output classes, whereas the car counting dataset has 65 output classes which predict car count from 0 to 64. Momentum was set at 0.9; the spikeDecay parameter which controls the backpressure of input spikes to a neuron was set at 7.5*e*−5; and weightDecay parameter was set at 1*e*−6 for all of the layers.

### 3.1. Accuracy Analysis

Table 2, shows the detection and accuracy for Alexnet (baseline neural network) and different CNN models that have been proposed in Figures 5A, 8A, 8B, 11A, 11B. The results of this table also quantifies the number of chips that are utilized to map the first three TN-deployed convolutional layers.

**Table 2:** Hardware resource analysis and testing accuracy for additional CNN structures.

**Model name**	**Detection accuracy (in %)**	**Counting accuracy (in %)**	**Chips required for first 3 TN CNN layers**
AlexNet	97.62	67.97	N/A
CNN Model 1 (Figure 13A)	97.87	68.62	19.88
CNN Model 2 (Figure 13B)	97.21	66.73	9.45
CNN Model 3 (Figure 13C)	97.52	68.21	8.67
CNN Model 4α (Figure 13D)	97.60	69.04	11.16
CNN Model 4β (Figure 13E)	90.98	53.4	11.16
CNN Model 5 (Figure 13F)	97.1	65.31	8.99

Based on the results reported in Table 1, a modified AlexNet model (Figure 5A) achieves significantly low accuracy compared to is floating-point counterpart (Figure 4A) that was implemented on a GPU. This loss in accuracy is due to ternary weight and binary activation representation that IBM TrueNorth computes on (as explained in McKinstry et al., 2018), as well as, aggressively downsampling the input images by a factor of 4 in the first layer because of which, the EEDN based CNN is not able to capture the unique features properly. Whereas, we can observe a significant improvement in accuracy with modified VGG-16 neural network models. Unlike AlexNet, the modified VGG-16 models (Figures 8A,B) are much deeper and are able to learn distinguishable features much more efficiently.

**Figure 12:**
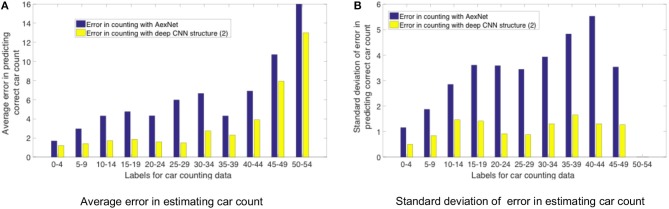
Error in estimating the label of car count vs the actual car count label. The plot compares the counting labels that were predicted with AlexNet CNN (Figure 5A) and deep modified VGG-16 model (Figure 11B). X-axis shows the range of labels associated with the counting dataset. For example, in the x-axis a value of 0–9 represents all of the counting dataset labels that were counting values in the range from 0 to 9. In **(A)** Y-axis plots the average error in estimating car count, and in **(B)** Y-axis plots the standard deviation of error in estimating car count.

Figure 12 shows a comparison between counting labels estimated by AlexNet CNN structure (Figure 5A) and deep modified VGG-16 model (Figure 12B) that were deployed on TrueNorth. As stated earlier, AlexNet model is not able to learn the distinguishable features as efficiently as the deeper CNN models. It can be observed from the plots in Figure 12 that average error is high for high value of counting labels. For high label values (45–49 and 50–54) images have high density of cars in them, therefore, it is important to have CNN structures that are able to learn the features which can detect individual cars and later use them for counting task.

**Figure 13:**
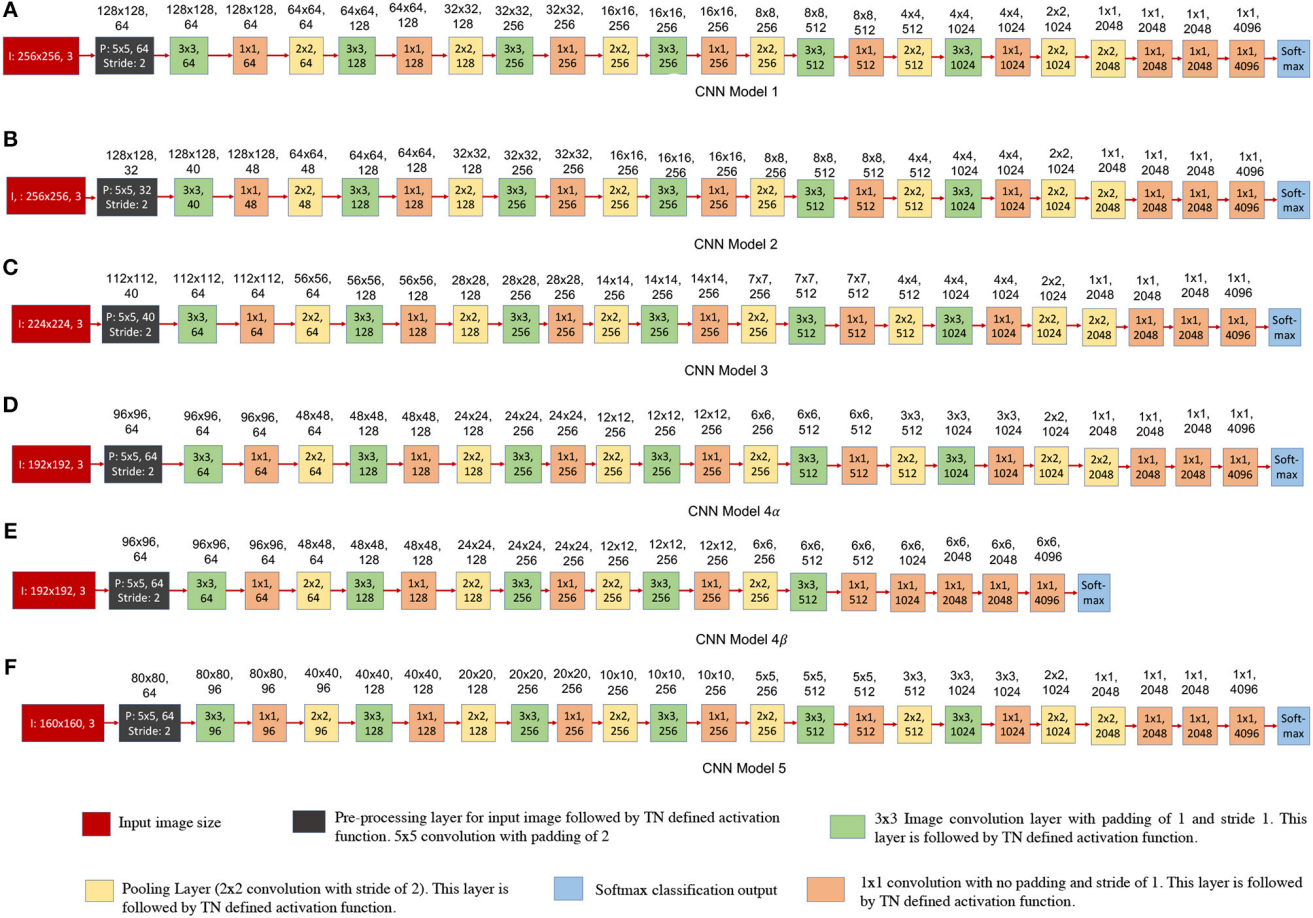
Convolutional neural network structures trained using EEDN for COWC dataset. The numbers written on top of the blocks show the output feature dimension of that block in CNN model. **(A–F)** shows different design decisions for all of the six CNN models. Each of the proposed CNN model either has (1) different input image size, or (2) different output feature count for first four convolutional layers, or (3) different number of pooling layers (CNN models 4α and 4β). **(A–D)** and **(F)** are all 23-layered CNN models, and the final layer serves as softmax loss function. **(D)** and **(E)** are meant for comparison with prior approach to model CNNs. **(E)** is a 19-layered CNN model, and in this structure we do not downsample the image features to a 1 × 1 patch.

Table 2, shows the detection and accuracy for Alexnet and different CNN models that have been proposed in Figure 13. The results of this table also quantifies the number of chips that are utilized to map the first three TN-deployed convolutional layers.

### 3.2. Experiments With Additional Neural Network Structures

Figure 13 shows the different CNN models that were trained using EEDN training algorithm. All of these proposed CNN models are a variation of deep CNN structure that was shown in Figure 11. Equation (1) shows the activation function used by CNN layers deployed on TN. It is important for us to understand how different input image size or feature count of convolutional layers would affect the hardware resource consumption and the test accuracy. If the CNN structure is designed naively, then we might waste critical compute resources for performing operations such as creating multiple instances of input data. On the other hand, if the proposed design is extremely conservative, then the accuracy may reduce significantly. Therefore, in this section we will discuss how different design proposals will affect hardware usage and dataset accuracy.

Each of the proposed CNN structure has a different input image size, and different output feature counts for the first four convolutional layers. The first convolutional layer (or transduction layer) is deployed on CPU/FPGA off-chip system (Esser et al., 2016; Sawada et al., 2016), whereas convolutional layers 2, 3, and 4 are deployed on the TrueNorth hardware. The proposed CNN models, Figures 13B–F require 16 chips to be deployed on TN hardware.

The CNN models shown in Figures 13A–D,F, are all 23-layered CNN models, and the final layer serves as softmax loss function. Figures 13D,E are meant for comparison with prior approach to model CNNs. Figure 13E is a 19-layered CNN model, and in this structure we do not downsample the image features to a 1 × 1 patch. Instead for CNN model 4β (Figure 13E) we downsample the patches until the size of the patch is 6-by-6 pixels. Even though CNN models 4α (Figure 13D) and 4β (Figure 13E) have a different number of layers, the input image size and feature count in the initial layers are the same for the both models

**Figure 14:**
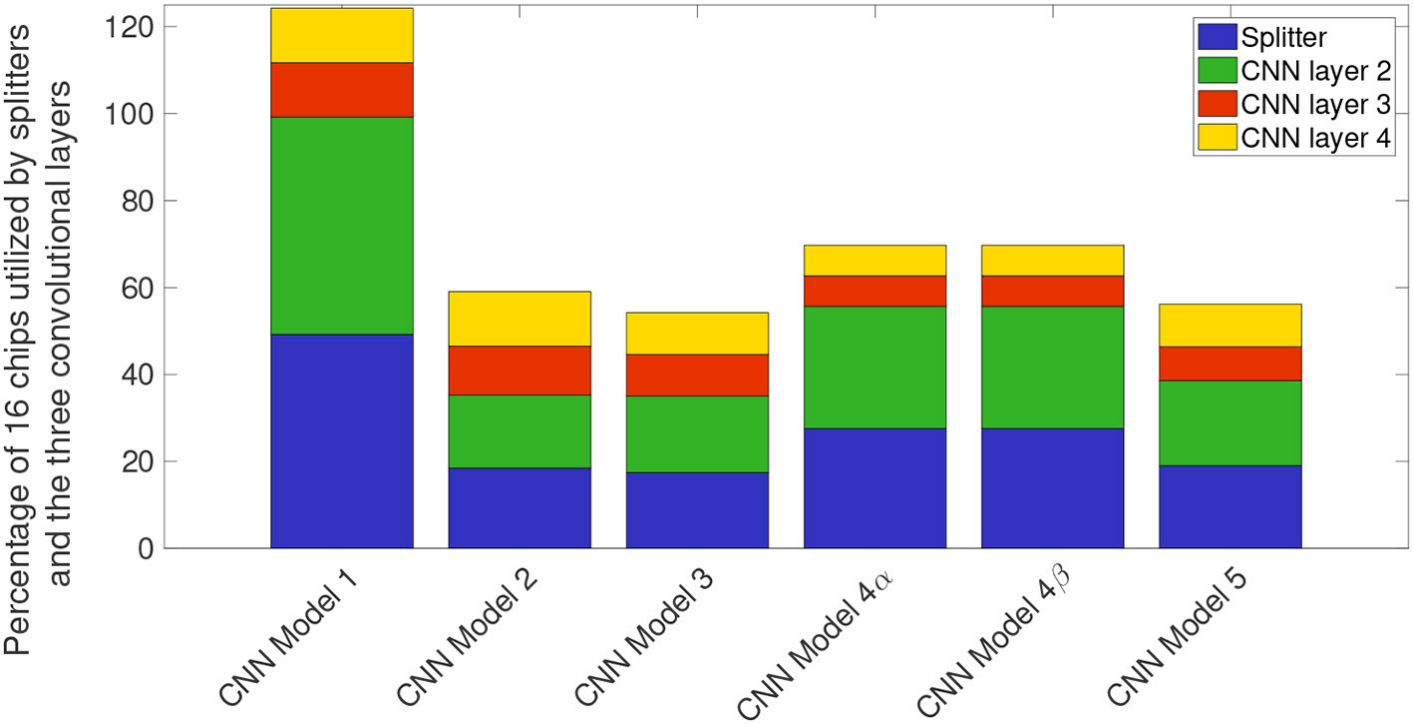
Stacked bar plot illustrating percentage chips utilized in the NS16e system by splitters and convolutional layers for different convolutional models.

Figure 14 shows the breakdown of chip utilization for the splitters, and convolutional layers 2, 3, and 4, since these four layers consumed the most number of hardware resources. In section 2.2.3.2 we introduced the concept of balancing input image size with the transduction layer's output feature count so that a minimum number of chips are used up for fan-out while keeping the test accuracy comparable to more standard approaches. Table 2 shows that by proposing a neural network architecture that is similar to CNN model 4, we can have test accuracy that is similar to the full precision AlexNet implementation. In CNN model 4 (Figure 13C), the input image is of size 192-by-192 pixels, as a result, there is no loss in pixel information due to early downsampling. If input images are downsampled aggressively (by using pooling layers), or the number of features is reduced significantly, test accuracy for detection and counting will also decrease. For example, if the input images are downsampled from 160-by-160 pixels to a small size of say 80-by-80 pixels in the first convolutional layer, then we can have more number of features, but the output accuracy is still less compared to CNN model 4. Having more output feature does not help in improving the test accuracy because the image features do not get captured nicely with an aggressive downsampling operation.

### 3.3. Comparison With Prior Approach

Section 2.2.3.6 motivated the need for fully convolutional neural networks where the image patch has been downsampled to a 1 × 1 patch. Prior work by Esser et al. (2016) proposed a fully convolutional neural network where a 64-by-64 pixel input image was downsampled to an 8-by-8 patch for output prediction. We compare our proposed CNN structure with the decision that was presented (Esser et al., 2016) and (Alom et al., 2018). We perform this comparison by analyzing the test accuracy of CNN model 4α (Figure 13D) and CNN model 4β (Figure 13E). In CNN model 4β, the input image patch is downsampled only to a 6-by-6 pixel patch. Both of these CNN models require 16 TN chips to be deployed. The training parameters were also the same for both of these models.

Based on the results shown in Table 2, we can observe there is a significant difference in test accuracy between the two models. This might be because CNN model 4β does not get to scan the entire image before making the prediction. In contrast, CNN model 4α is able to find a relationship between all of the pixels in the image and provide a better output prediction. There is a difference of 6.62% in detection accuracy and 15.64% in counting accuracy between CNN model 4α and CNN model 4β, with our approach of CNN model 4α having a considerably higher test accuracy.

### 3.4. Hardware Analysis

As per the detection and counting accuracies shown in Table 2, CNN model 4α (Figure 13C) has the best accuracy among all of the neural network models that were evaluated. This model can also be deployed on NS16e TrueNorth hardware. Therefore, rest of the discussion in this section will focus on the test accuracy results obtained from CNN model 4, as well as report hardware analysis for this neural network model.

Table 2 shows the results for COWC dataset after the trained network (CNN model 4) was deployed on NS16e system. Neural network structures for both counting and detection tasks consumed all of the 16 chips available in NS16e platform. The standard neural networks were implemented using the Caffe neural network framework (Jia et al., 2014) and the trained full-precision neural networks were deployed on NVIDIA Titan X GPU. Table 3 shows the percentage accuracy for three different tasks. The first task is car detection, a binary classification problem where the goal is to predict whether a car has been detected in the center of the image or not. For the entire detection test dataset, accuracy of car detection with CNN model 4 (Figure 13C) is 97.35%, precision score is 96.36%, recall score is 97.33%, and the F1 score of this task is 96.84%. Overall, the mapped neural network on TrueNorth does very well in detecting the objects. The second task is to count the number of cars in the image and predict how many cars are present in the image in the range from 0 to 64. The third goal is to count the number of cars in the image by relaxing the output prediction condition; that is, if an error margin of −/+ 2 is allowed for estimating the car count, then what would be the prediction accuracy. For example, in Figure 1C the correct label is 13 for counting. With −/+ 2 margin error, if the neural network predicts any label in the range ∈ [11, 15] our model would classify that as a correct output with respect to the input image of Figure 1C.

**Table 3:** This table reports accuracy for car detection and car counting on TrueNorth and NVIDIA Titan X, as well as throughput for car counting on these platforms.

**Neural network structure**	**Detection accuracy (in %)**	**Counting accuracy (in %)**	**Counting accuracy with −/+2 error margin (in %)**	**Frames per second (FPS) for counting task**	**FPS per watt**	
Truenorth (EEDN)	97.60	69.04	96.57	3.38	0.444 (at 0.775 V)	0.387 (at 1.0 V)
AlexNet	97.62	67.97	98.82	11.48	0.046	
GoogLeNet	99.12	80.35	98.87	2.73	0.011	
ResCeption	99.14	80.34	98.86	2.91	0.012	

As per the results for CNN model 2 (Figure 13C) in Table 3, neural networks deployed on TrueNorth with EEDN framework have accuracy that is close to AlexNet, but have a considerable difference when compared with GoogLeNet (as proposed in Szegedy et al., 2014) and ResCeption (as proposed in Mundhenk et al., 2016). This could be due to the rich feature representations that GoogLeNet and ResCeption can capture. Each layer in these two neural networks has different-sized filters operating in parallel, and the outputs from these filters get depth concatenated. As a result GoogLeNet and ResCeption can capture robust, differentiable features. However, this difference reduces significantly with an allowed error margin of −/+ 2 when predicting the car count.

Table 3 shows the frames per Second (FPS) for car counting based classification problem for the neural networks that were deployed on different hardware platforms. As per (Mundhenk et al., 2016) a single frame in FPS is defined as the scene of size 2048-by-2048 pixels with additional padding so that the first patch has a center at (0,0). The image frame is divided into multiple patches of 192-by-192 pixels and a stride of 167 pixels. Therefore, the frames per Second (FPS) for counting task is meant to quantify how fast the CNN models can scan though an entire image frame of 2048-by-2048 pixel and be able to count the number of cars in this entire frame. The tick period of TrueNorth operation had to be increased to 1.75 ms (operating frequency was reduced to 571.43 Hz) to get the results shown in Table 3, possibly because for smaller tick period, spikes were getting bottlenecked when trying to cross chip boundaries. Article on TrueNorth ecosystem (Sawada et al., 2016) presents how spikes travel during inter-chip communication. First a spike has to traverse one row of the network-on-chip, then travel through the chip I/O peripheral circuitry and finally it is delivered to the destination chip through limited I/O connections that are present between two chips. Since the spikes have to travel peripheral circuitry and limited I/O connections that are present between two chips, these sections become a bottleneck for inter-chip communication if the spike rate is high. As a result, the spikes were not getting delivered for smaller tick periods since the inter-chip communication bandwidth was becoming the bottleneck for multi-chip networks. Prior work by Akopyan et al. (2015) have proposed wire-length minimization placement algorithm for TrueNorth. A better placement of cores could improve the runtime as well as the FPS.

In this section we report the first-order analysis of NS16e TrueNorth power consumption values based on the analysis that was presented in Merolla et al. (2014) and Sawada et al. (2016). TrueNorth chips can operate at 0.775 V and 1.0 V. The power consumption values were calculated with an operating frequency of 571.43 Hz, static power was set to 70 mW for 0.775 V operating voltage and 114 mW for 1.0 V operating voltage. We assumed that dynamic power is the same as static power for an operating frequency 1KHz and later these dynamic power values were scaled down linearly to account for the chip operating frequency of 571.43 Hz. When all of the chips on NS16e board are computing at the same time, the total combined active power consumed by TrueNorth chips is 1.76 W and 2.87 W with the operating voltage set at 0.775 V and 1.0 V, respectively. Total peak power consumed by the NS16e system is 7.62 W for 0.775 V operating voltage and 8.73 W for 1.0 V operating voltage. In contrast, an NVIDIA Titan X GPU can consume a peak power of 250 W to run these neural network structures at its highest frames per second rate.

## 4. Discussion

### 4.1. Summary

In this paper we described four design decisions that a designer would have to address to deploy CNN structures on a neurosynaptic system such as IBM TrueNorth. These decisions are very important if the goal is to perform tasks such as detection and counting in a hardware constrained environment. Section 2.2 introduced the need to have a systematic approach for proposing neural network designs that can be mapped onto TrueNorth. Here we discussed how we can leverage prior work that have been proposed for CNN design and extend those ideas to EEDN based CNN models for TrueNorth. We showed that if a standard VGG-16 CNN model is modified systematically, while keeping in mind the architectural bottlenecks that are present in NS16e, hardware resource requirements can be reduced by 3x (refer to Figures 9, 10).

Similarly, we discussed in Table 1 that with systematic approach to mapping CNNs on TrueNorth, the accuracy could be improved by 8% for detection based task and by 20% for counting based task when compared to having a naive ternary-weight AlexNet implementation on NS16e. Results presented in Table 2 show that EEDN trained neural network can have similar accuracy as full precision AlexNet.

It is important for us to consider how many TN cores are performing relevant computations. The analysis presented in Figure 14 shows that it is extremely important for users to consider the trade-off between the hardware resources that is available for mapping the neural network, and the input image size and feature counts of initial layers, to achieve the desired test accuracy.

Section 3.4 analyzes the cost of the deployed neural network on TN hardware. As per the results presented in Table 3, the EEDN-trained neural network when deployed on TN hardware has test accuracy that is comparable to high-precision neural networks like AlexNet, GoogLeNet, and ResCeption, but shows a manifold improvement in FPS per watt.

### 4.2. Extending This Work to Other Benchmarks and Neuromorphic Chips

As neuromorphic computing is becoming more promising, it is important for researchers to understand the challenges that came up in TrueNorth architecture/algorithm and address these issues in future neuromorphic computing architectures/algorithms.

First, it is important for us to have a new set of benchmarks and datasets that can be used to evaluate neuromorphic hardware for bigger CNN models or that require us to estimate continuous numbers such as regression problems. There have been benchmarks that were proposed keeping in mind SNN algorithms, viz., N-MNIST (Orchard et al., 2015) and CIFAR-10 DVS (Li et al., 2017), but both of these benchmarks have very small image sizes and both of these benchmarks can solved using classification models. Problems that require us to estimate continuous numbers bring out the architectural limitations that might arise if the goal is to predict large range of numbers. On the other hand, benchmarks from domains such as Micro-Aerial Vehicles (Ma et al., 2013) and video surveillance would be very interesting for the SNN community because these small drones already have SNN controllers in them (Clawson et al., 2016). Having video surveillance dataset from MAVs, will help us realize potential of SNNs to be deployed in energy-constrained environments. Evaluating the hardware with bigger CNN models will help us understand the architectural limitations that are present in the hardware and it will also motivate researchers to investigate better algorithms for hardware/software co-design on neural networks.

Second, it is critical to investigate the fan-out limitations of architectures such as TrueNorth, so that neural networks can also support connections between convolutional and fully-connected layers. Even though there have been prior research that have proposed algorithms to train inception neural networks or residual networks for SNN hardware (Rueckauer et al., 2017; Sengupta et al., 2018), the current architectural limitations related to fan-out in SNN hardware such as TrueNorth, do not support such skip connection based CNNs. Concurrently, CNN structures such as MobileNets (Howard et al., 2017) have shown to significantly reduce the memory accesses and computations for embedded platforms. To the best of author's knowledge, currently there is no research that has successfully trained ternary quantized model for depthwise separable filters, which is a critical part of MobileNets. Prior work done in Holesovsky and Maki (2018) have attempted to train a depthwise separable CNN with ternary weights and activation, but reported a significant drop in accuracy when compared to the same CNN structure that was trained with single precision weights and activation.

Third, it is important to address the architecture bottlenecks present between the CPU/FPGA hybrid system and the neuromorphic chips, otherwise, a considerable amount of computation resources may end up getting used up to handle these interactions, as shown in CNN baseline example of Figure 14. Another direction that researchers can potentially investigate is improving the speed of deployed neural networks by analyzing the bottleneck present during inter-chip communication on a scaled-up hardware such as NS16e system.

Finally, as neural network models become deeper and wider, there will be a considerable amount of communication happening between neurons mapped onto different chips. This bottleneck could be addressed by having a better placement algorithm for multi-chip placement which would constrain group neurons that communicate a lot with each other to a single chip, unlike the work proposed in Akopyan et al. (2015) where the goal of the placement algorithm is to minimize the wire-length of placed neurons. Or, researchers can propose a new interconnect architecture for inter-chip communication that could handle high backpressure of spikes that get delivered from one neuromorphic chip to another.

Pruning may not always be the best approach to address hardware constraints while DNN training. As presented in Yazdani et al. (2018) even though pruning may give correct test accuracy, the inference confidence score reduces significantly. Researchers from hardware community have proposed pruning algorithms to reduce the size of bigger CNNs for hardware deployment (Han et al., 2015; Iandola et al., 2016). At present EEDN trained CNN models are highly sparse due to ternary weight representation, having more aggressive, such as pruning away TN cores for deep learning model, pruning technique may result in further drop in test accuracy. Therefore, rethinking the placement strategy for deep learning models on SNN may be an important step forward to address the issue of hardware constraints.

## Author Contributions

RS was the one that led this project. He came up with the idea, suggested the plan of execution, performed all of the experiments and wrote this paper. ML, BV, AM, and NM provided feedback for the work that RS did and also gave suggestions about how to improve the manuscript.

### Conflict of Interest Statement

ML has financial interest in Thalchemy corp. and is co-founder of the said corporation. Thalchemy corp. was not at all involved in this research project in any form. The remaining authors declare that the research was conducted in the absence of any commercial or financial relationships that could be construed as a potential conflict of interest.
